# Numerical Investigation of Swimmer’s Gliding Stage with 6-DOF Movement

**DOI:** 10.1371/journal.pone.0170894

**Published:** 2017-01-26

**Authors:** Tianzeng Li, Wenhao Cai, Jiemin Zhan

**Affiliations:** Department of Applied Mechanics and Engineering, Sun Yat-sen University, Guangzhou, China; Nanyang Technological University, SINGAPORE

## Abstract

The purpose of this study is to analyze the motion status of swimmers during their gliding stage using a numerical simulation method. This simulation strategy is conducted by solving the 3D incompressible Navier-Stokes equations using the Realizable k-ε turbulence closure equations in combination with the Six Degrees of Freedom (6-DOF) method. The uneven mass distribution of a swimmer and the roughness of the surface of the body are taken into consideration. The hydrodynamic characteristics and movement characteristics of the swimmers at different launch speeds were analyzed. The calculated results suggest that an optimal instant for starting propulsive movement is when the velocity of the swimmer decreases by 1.75 m/s to 2.0 m/s from an initial horizontal velocity of 3.1 m/s to 3.5 m/s.

## Introduction

Gliding underwater, which is a major part of the overall performance in competitive swimming, occurs during the starts, between strokes, and after turns [1,2]. A proper gliding posture can offer relatively higher initial velocity and can maintain the momentum of the swimmer for the next stroke, and the proper gliding posture leads to an increase in the overall swimming rhythm.

The average velocity of the glide stage, commonly used to assess the glide performance of swimmers, is determined using the initial velocity, the rate of speed loss of the swimmers, and the glide duration [3]. The initial velocity depends on the preceding actions, including take-off and entry. Glide performance can be improved by increasing the initial velocity of the swimmer, but increasing the initial velocity of the swimmer will lead to increasing the metabolic cost. Improving the gliding efficiency (the ability of the swimmers to minimize deceleration during the gliding stage) can be beneficial because improving the gliding stage is an advantage that is gained without extra metabolic cost [1]. The velocity of swimmers will gradually decrease as the hydrodynamic drag (including form drag, friction drag and wave drag) acts on the swimmers during underwater gliding. The hydrodynamic drag of the swimmer is affected by the different factors which include human morphology, gliding speed and depth et al. [4]. Researchers [5, 6, 7] have carried out several studies about the effect of depth and velocity on drag which intended to define the appropriate glide depths and velocities to decrease wave drag. The effective streamline postures (e.g. the prone streamline glide, the lateral, streamline glide or even body rolling) during the underwater gliding motion can gain less form drag so that improves swimming performance [8, 9]. In addition, an optimal instant to initiate underwater leg propulsion plays a crucial role in the swimming start and after turns [10]. Too long or too short of a glide duration is unfavorable for swimming performance. Studies [8, 11] have shown that a proper instant of initialization of underwater leg movement for a grab start is that the swimmers’ glide velocity is close to the velocity of the underwater leg propulsion (approximately 2.2 to 1.9 m/s) at which to begin underwater kicking to prevent energy loss from excessive active drag.

Experimental approaches have played a major role in the study of the gliding stage over the past decades [8, 10, 11]. Significant efforts have been undertaken to understand swimming mechanics on a deeper basis [12]. However, for most experimental research, the researchers generally emphasize the analysis of experimental data and lack a persuasive explanation for a phenomenon due to the limitations of the experiments. In recent years, a new approach, numerical simulation, has arisen with the development of computer technology. The numerical simulation method has been extensively applied in the study of the passive drag of swimmers during underwater gliding as it can provide a visualization for the flow field and can evaluate different components of the drag force [13]. For example, Popa et al. [14] conducted a series of numerical studies about the influence of postural change of the swimmer's head in hydrodynamic performances. Novais et al. [15] have researched the effect of water depth to hydrodynamic drag using numerical simulation method, which shows that hydrodynamic drag decreased with depth, although after 0.75m values remained almost constant. Marinho et al.’s numerical study [16] shows that the position with the arms extended at the front presented lower drag values than the position with the arms aside the trunk, etc. Nevertheless, the swimmer is generally placed in a constant position in the flume with a stationary posture for most of current numerical investigations of swimming gliding, which cannot represent the actual gliding motion of a swimmer. Numerical studies of motion characteristics during underwater gliding are scarce.

In our study, we aim to reveal the process of the gliding motion and to analyze the hydrodynamic characteristics of swimmers using a numerical simulation method. The numerical simulation strategy is carried out by solving the 3D incompressible Navier-Stokes equations using the realizable *k* − *ε* turbulence model with consideration of the six degrees of freedom (6-DOF) movement. A set of cases with different initial velocities are studied. Swimmers’ drag force, movement characteristics and vortex structure, etc., are analyzed. Numerical results are utilized to verify the rationality of the traditional optimal instant to initiate underwater leg propulsion.

## Materials and Methods

### Numerical approach

#### Governing equations

The flow of this simulation is assumed to be an incompressible viscous fluid, described by the continuity equation and the Navier–Stokes equations including the mass and momentum conservation equations as follows:

$$\frac{\partial }{\partial x_{i}}\left(u_{i}\right)=0$$ (1)

$$\frac{\partial }{\partial t}\left(u_{i}\right)+\frac{\partial }{\partial x_{j}}\left(u_{i}u_{j}\right)=-\frac{1}{\rho }\left(\frac{\partial p}{\partial x_{i}}\right)+g_{i}+\frac{\partial }{\partial x_{j}}\left(\nu \frac{\partial u_{i}}{\partial x_{j}}\right)$$ (2)

where *ρ* is fluid density, *u*_*i*_ and *u*_*j*_ are the components of the velocity vector, *p* is the pressure, *g*_*i*_ is the component of the gravitational acceleration, *ν* is the kinematic viscosity, and the index *i*,*j* = 1,2,3 for three dimensional flows.

The governing Navier-Stokes equations are time averaged under the Reynolds-averaged Navier-Stokes (RANS) framework, and the generated fluctuation terms are modeled with the introduction of two new terms, turbulent kinetic *k* and dissipation rate *ε*. In this study, the two new variables are resolved by the realizable *k* − *ε* model, which performs better than other RANS models for separated flows and flows with complex secondary flow features [17].

$$\frac{\partial }{\partial t}\left(\rho k\right)+\frac{\partial }{\partial x_{j}}\left(\rho ku_{j}\right)=\frac{\partial }{\partial x_{j}}\left[\left(\mu +\frac{u_{t}}{\sigma_{k}}\right)\frac{\partial k}{\partial x_{j}}\right]+G_{k}+G_{b}-\rho \varepsilon -Y_{M}+S_{k}$$ (3)

$$\frac{\partial }{\partial t}\left(\rho \varepsilon \right)+\frac{\partial }{\partial x_{j}}\left(\rho \varepsilon u_{j}\right)=\frac{\partial }{\partial x_{j}}\left[\left(\mu +\frac{u_{t}}{\sigma_{\varepsilon }}\right)\frac{\partial \varepsilon }{\partial x_{j}}\right]+\rho C_{1}S\varepsilon -\rho C_{2}\frac{\varepsilon^{2}}{k+\sqrt{\nu \varepsilon }}+C_{1\varepsilon }\frac{\varepsilon }{k}C_{3\varepsilon }G_{b}+S_{\varepsilon }$$ (4)

where $C_{1}=\max\left[0.43,\frac{\eta }{\eta +5}\right]$, $\eta =S\frac{k}{\varepsilon }$ and $S=\sqrt{2S_{ij}S_{ij}}$. *G*_*k*_ is the generation of turbulence kinetic energy due to the mean velocity gradients, and *G*_*b*_ is the generation of turbulence kinetic energy due to buoyancy. *Y*_*M*_ is the contribution of the fluctuating dilatation in compressible turbulence to the overall dissipation rate. *C*_2_ and *C*_1*ε*_ are constants, 1.9 and 1.44, respectively. $C_{3\varepsilon }=\tanh\left|\frac{v}{u}\right|$, *ν* is the component of the flow velocity parallel to the gravitational vector and *u* is the component of the flow velocity perpendicular to the gravitational vector. *σ*_*k*_ and *σ*_*ε*_ are the turbulent Prandtl numbers for *k* and *ε* as 1.0 and 1.2, respectively. *S*_*k*_ and *S*_*ε*_ are user-defined source terms.

The swimming simulation is conducted in a 3D flume with a two-phase flow. Water and air are specified by a free surface. The volume of fluid (VOF) method is applied to track the deformation of the air-water interface and has been successfully applied in our previous numerical studies of swimming [18, 19]. The standard wall function which is a semi-empirical formula to correlate the variables in near-wall region and relevant variables in fully-turbulent region is used to resolve the flow near the swimmer’s surface

$$U^{*}=\frac{1}{\kappa }\ln\left(Ey^{*}\right)$$ (5)

where, U* and y* represents the dimensionless velocity and the dimensionless distance from the wall, respectively. *κ* is the Von Karman constant (recommended value = 0.4187) and *E* is the empirical constant (recommended value = 9.793).

#### Dynamic mesh theory

In this numerical simulation, the dynamic mesh method is applied to reveal the motion of a swimmer. The meshes of the domain are changed with time with the motion of the boundaries of a swimmer by the dynamic mesh method. The integral form of the conservation equation for a general scalar *ϕ*, on an arbitrary control volume *V*, whose boundary is moving can be written as

$$\frac{d}{dt}\int_{V}\rho \phi dV+\int_{\partial V}\rho \phi \left(u-u_{g}\right)\cdot dA=\int_{\partial V}\Gamma \nabla \phi \cdot dA+\int_{V}S_{\phi }dV$$ (6)

where *ρ* is the fluid density, ***u*** is the flow velocity vector, ***u***_*g*_ is the mesh velocity of the moving mesh, Γ is the diffusion coefficient, and *S*_*ϕ*_ is the source term of *ϕ* [17].

The swimmer is set as a rigid body. The motion of the body can be described by six degrees of freedom (6-DOF)—three translational degrees of freedom of the mass center (surge, sway and heave) and three independent rotational degrees of freedom of the relative three axes (pitch, roll and yaw) [20]. The governing equation for the translational motion of the center of the swimmer’s gravity is solved in the inertial coordinate system:

$${\dot{v}}_{G}=\frac{1}{m}\sum f_{G}$$ (7)

where ${\dot{v}}_{G}$ is the translational motion of the center of gravity, *m* is the swimmer’s mass, and ***f***_*G*_ is the force vector relative to the global coordinate system.

The angular movement of the swimmer can be determined using body coordinates. The dominant equation of this movement is:

$${\dot{\omega }}_{B}=L^{-1}\left(\sum M_{B}-\omega_{B}\times L\omega_{B}\right)$$ (8)

where *L* is the inertia tensor, ***M***_*B*_ is the moment vector of the body, and ***ω***_*B*_ is the rigid body angular velocity vector.

The mesh updating strategy in the deformable mesh zone over time uses a coupling of spring-based smoothing and the local cell re-meshing method [17].

#### Boundary conditions

The 3D numerical flume simulates a widely used lane of swimming pool in Chinese universities with the width of 2.5 m and the depth of 1.8 m. The swimmer is positioned horizontally in the left side of the flume. In order to guarantee sufficient gliding distance for swimmer model, the longitudinal length of the flume is set as 15 m. The thickness of the air layer above the water surface is 1.2 m. The perpendicular distance of the swimmer’s initial center of gravity from the surface of the water is 0.9 m, where the effect of wave drag on the swimmer can be neglected [4]. The top of the flume is set as a pressure outlet condition with a constant value of standard atmospheric pressure. In general, the bottom of pool is paved with tiles which have glossy surface. Thus, a non-slip wall condition with zero roughness is imposed at the bottom of the current numerical flume. Additionally, symmetrical boundary conditions where velocity and gradients of all variables are zero are applied around the flume as recommended by previous studies [21, 22].

In this study, the swimmer is assumed to be a rigid body. The surface of the swimmer is set as a non-slip wall condition. The roughness of the surface is considered as equivalent to sand-grain roughness by modifying the law-of-the-wall for turbulent wall-bounded flows. The law-of-the-wall for the mean velocity in the wall function is modified as follows:

$$\frac{u_{p}u^{*}}{\tau_{w}/\rho }=\frac{1}{\kappa }\ln\left(E\frac{\rho u^{*}y_{p}}{\mu }\right)-\Delta B$$ (9)

$$u^{*}=C_{\mu }^{1/4}k^{1/2}$$ (10)

$$\Delta B=\frac{1}{\kappa }\ln f_{\gamma }$$ (11)

where *f*_*γ*_ is a roughness function involving roughness effects, and Δ*B* is closely related to the nondimensional roughness height $K_{s}^{+}$.

$$K_{s}^{+}=\rho K_{s}u^{*}/\mu$$ (12)

where *K*_*s*_ is physical roughness height. In general, the status of the surface roughness can be divided into three distinct regimes according to the $K_{s}^{+}$ value, e.g., hydrodynamically smooth ($K_{s}^{+}\leq 2.25$), transitional ($2.25\leq K_{s}^{+}\leq 90$) and fully rough ($K_{s}^{+}>90$). In many previous numerical studies [13, 14], the roughness of the swimmer’s surface is assumed to be zero as it satisfies the condition of a hydrodynamically smooth flow regime for the velocity range from 1.4 m/s to 3.1 m/s. However, in the present study, the mean physical roughness height of the healthy Chinese male (approximately *K*_*s*_ = 2.4 × 10^−5^ m) is applied in the initial velocity range from 3.1 m/s to 3.5 m/s. The swimmer’s surface roughness manifests mainly in the transitional status. Therefore, the effect of roughness on the swimmer is considered in the simulation. The equation proposed by Cebeci and Bradshaw based on Nikuradse’s data [23] can be defined as follows:

$$\Delta B=\frac{1}{\kappa }\ln\left[\frac{K_{s}^{+}-2.25}{87.75}+C_{s}K_{s}^{+}\right]\times \sin\left[0.4258\left(\ln K_{s}^{+}-0.811\right)\right]$$ (13)

where *C*_*s*_ is a roughness constant that depends on the type of roughness. *C*_*s*_ is set as 0.5 in this study.

#### Numerical implementation

The simulation strategy with the 3D incompressible Navier-Stokes equations is integrated using commercial computational fluid dynamic software, ANSYS FLUENT V15.0 [17]. The Pressure Staggering Option (PRESTO) discretization scheme was used to calculate pressure. The Quadratic Upwind Interpolation of Convective Kinematics (QUICK) algorithm was used to compute the convection terms, and the pressure implicit with splitting of operators (PISO) algorithm was used for the pressure-velocity coupling.

### Physical parameters of the swimmer model

**Fig 1:**
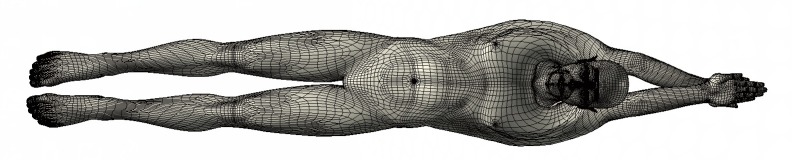
3D model of the swimmer with a traditional streamlined posture.

In our study, a 3D virtual human model based on the mean anthropometrical characteristics of the high-level Chinese male swimmers is created by Computer Aided Industrial Design (CAID) software (Rhinoceros v5.0, Robert McNeel & Associates, USA), which has a powerful model-building capacity. The traditional streamlined posture that is characterized by an elongated posture with arms extended forward with hands pronated and overlapping was investigated, as shown in Fig 1.

**Fig 2:**
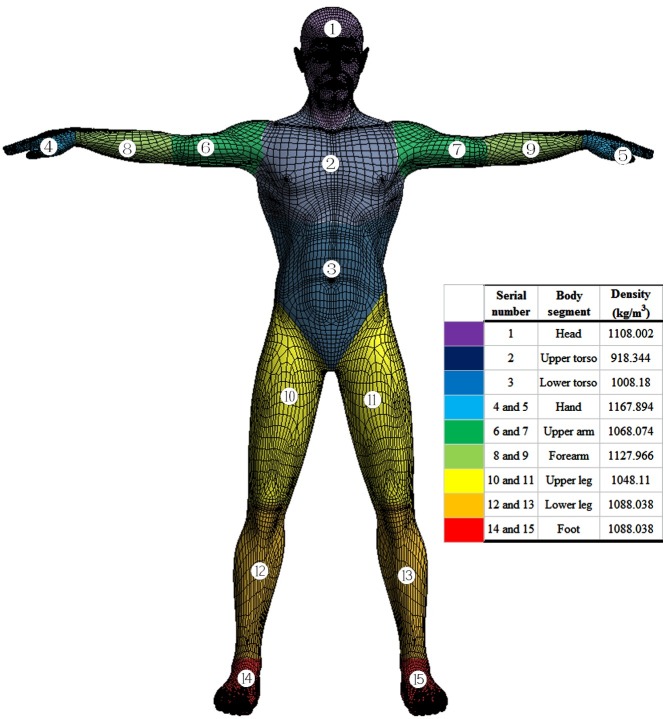
Segment model of human body.

As our current study of the swimmer’s glide is an unprescribed motion based on 6-DOF movement, the subsequent motion of the swimmer is determined by the solution at the current time. The physical parameters of the swimmer (e.g., mass and moment of inertia) should be defined beforehand. Due to the difference in the distribution of skeletons, muscles and fat, etc., the density of each part of the human body varies. However, we notice that in some parts of the human body, the density is almost the same. The human body could therefore be separated into several segments according to the density distribution. The density in any segment is a constant, whereas among those segments, the density varies. In this paper, the human body is separated into 15 segments [24], as shown in Fig 2. The whole body can be assumed to be an integration domain Ω and to be composed of 15 subdomains Ω_1_,Ω_2_……Ω_*n*_. The integral value of Ω could be obtained through summing the values on each subdomain after completing the integration process on each subdomain. Thus, the moment of inertia of the whole body around a three-dimensional axis *l* can be calculated as follows:

$$I=\underset{\Omega_{1}}{∭}\rho r^{2}\text{d}V+\underset{\Omega_{2}}{∭}\rho r^{2}\text{d}V+\ldots \ldots +\underset{\Omega_{15}}{∭}\rho r^{2}\text{d}V=I_{1}+I_{2}+\ldots \ldots I_{15}$$ (14)

where Ω = Ω_1_∪Ω_2_∪……∪Ω_15_, *ρ*(*x*,*y*,*z*) is density distribution for each subdomain, *r*(*x*,*y*,*z*) is the distance from any point on the rigid body to the axis *l*, and *I*_1_,*I*_2_,……*I*_*n*_ represents the moment of inertia of each part of the rigid body.

In general, it is difficult to obtain the moment of inertia of a rigid body around a given axis. However, it will become easy when the axis crosses through the object's center of gravity. In our model, the mass *m*_*i*_ of each body segment, the centrobaric coordinate *x*_*i*_,*y*_*i*_,*z*_*i*_ of each segment, and the moment of inertia *I*_*xi*_,*I*_*yi*_,*I*_*zi*_ of each segment around the corresponding centrobaric axis could be calculated using the modeling software, Rhinoceros v5.0. According to the parallel axis theorem [25], the moment of inertia *I*_*x*_,*I*_*y*_,*I*_*z*_ of whole human body could be expressed as follows:

$$\left\{ \begin{array}{l} I_{x}=\sum_{i=1}^{15}I_{xi}+\sum_{i=1}^{15}m_{i}\left[{\left(y-y_{i}\right)}^{2}+{\left(z-z_{i}\right)}^{2}\right]\\ I_{y}=\sum_{i=1}^{15}I_{yi}+\sum_{i=1}^{15}m_{i}\left[{\left(x-x_{i}\right)}^{2}+{\left(z-z_{i}\right)}^{2}\right]\\ I_{z}=\sum_{i=1}^{15}I_{zi}+\sum_{i=1}^{15}m_{i}\left[{\left(x-x_{i}\right)}^{2}+{\left(y-y_{i}\right)}^{2}\right] \end{array}\right.$$ (15)

where the centrobaric coordinate *x*,*y*,*z* of whole human body can be defined by following formula:

$$\left\{ \begin{array}{l} x=\frac{1}{m}\sum_{i=1}^{15}x_{i}m_{i}\\ y=\frac{1}{m}\sum_{i=1}^{15}y_{i}m_{i}\\ z=\frac{1}{m}\sum_{i=1}^{15}z_{i}m_{i} \end{array}\right.$$ (16)

The main physical parameters of the swimmer for this simulation are listed in Table 1.

**Table 1:** Main physical parameters of the swimmer.

	Value	Unit
Mass	81.87	Kg
Height	1.82	m
Upper extremity length	0.80	m
Lower extremity length	0.91	m
Shoulder breadth	0.42	m
Pelvis breadth	0.34	m
Cheat circumference	0.98	m
Waist circumference	0.79	m
Hip circumference	0.92	m
Thigh circumference	0.58	m
Crus circumference	0.38	m
Volume	0.08	m^3^
Surface area	1.93	m^2^
The length from toe to finger (*L*)	2.43	m
Moment of inertia for centroid coordinate X-axis	0.90	kg·m^2^
Moment of inertia for centroid coordinate Y-axis	19.61	kg·m^2^
Moment of inertia for centroid coordinate Z-axis	20.01	kg·m^2^

## Validation of Model

**Fig 3:**
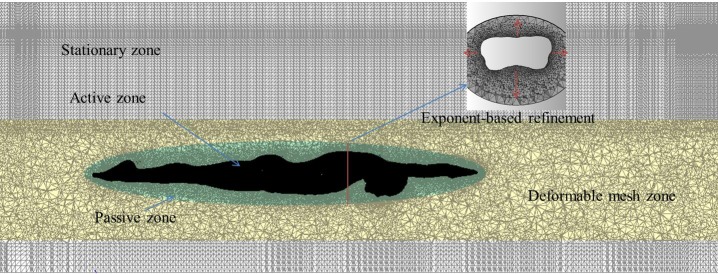
Sketch of computational mesh.

**Fig 4:**
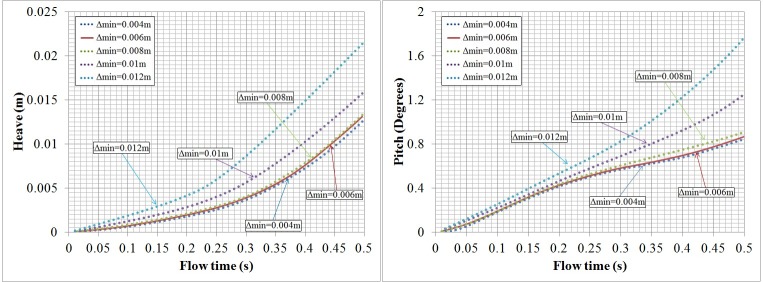
Swimmers’ pitch and heave with time for different grid resolutions.

In the 6-DOF simulation strategy, the meshes of the computational domain should be defined as different functional parts, namely, an active zone that corresponds to the boundary on which the loads are calculated and that delimits a body with defined dynamic characteristics, a passive zone that surrounds and moves with the active zone with non-deformation, a deformable zone where meshes are deforming with time, and constituted by tetrahedral types [26]. Additionally, a stationary zone that is outside the deformable mesh zone is set with structured hexahedral cells to save computing resources, as shown in Fig 3. To investigate the grid sensitivity, the computational mesh is refined using an exponent-based refinement method near the model’s surface with different mesh resolution, i.e., the smallest grid size near the surface is generated as 0.004 m, 0.006 m, 0.008 m, 0.0 1 m and 0.012 m. Cases with different grids are conducted with an initial velocity of 3.5 m/s. Swimmers’ heave and pitch values within the first 0.5 s are compared, as they are sensitive to the grid resolution, as shown in Fig 4. The results were insensitive to further reductions in resolution as the minimum wall normal grid spacing was reduced beyond 8 mm. As a result, the grid resolution of 0.006 m with approximately 7,500,000 cells is conservatively applied in the following studies.

**Fig 5:**
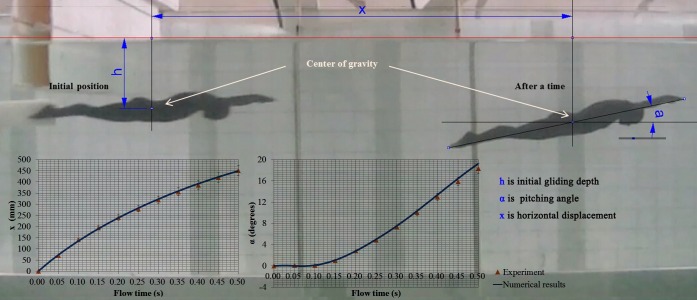
Validation of 6-DOF method using a scaled swimmer model with a streamlined posture.

**Fig 6:**
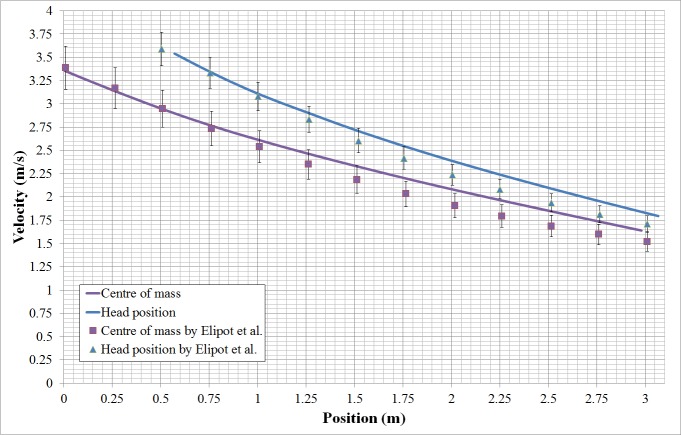
Comparison of the numerical results with the experimental ones by Elipot et al. for the variation of swimmers’ surge with position.

In the early stages of this study, we conducted a numerical and experimental investigation of 6-DOF scaled-down model of a swimmer posed in streamlined posture for validating the feasibility of our numerical strategy using a 6-DOF method. The scaled model which is 1/12.5 times of prototype is positioned at a depth of 60mm from the water surface and catapulted at a scaled initial velocity of 1.65m/s. High speed camera records the entire sequence of gliding movement with 200 frames per-second. The displacements and attack angles of the model are extracted from the snapshots captured by the camera within 0.5 second after being catapulted. By comparison, the results of numerical strategy using the 6-DOF method are in good agreement with the experimental data, as shown in Fig 5. However, in the study of the scale-down model, the material of the scale-down model is homogeneous without considering the different density of each body part. Additionally, surfaces of body were assumed to be smooth. Thus a case which is closer to reality (e.g. considering full-scale boy, density distribution of each part, roughness of surface and so on.) should be validated. Elipot et al. [10, 11] conducted an experimental analysis of swimmers’ velocity during underwater gliding motion following a graded start by testing eight high-level swimmers. A similar case was simulated using our current numerical strategy and validated compared with the experimental results by Elipot. Fig 6 shows the comparison of the numerical results with the experimental results for the variation in the swimmers’ surge with position. In the initial stage of the glide, the numerical results agree well with the experimental data. However, numerical results have higher velocity than experimental data with the increasing displacement of the swimmer. Our model is not exactly the same as the real swimmers, which is used in Ref. [10, 11]. Additionally, real swimmers cannot maintain a completely consistent posture over the glide stage. Therefore, the difference between the simulation and the numerical value is inevitable. However, the tendency toward variation of the calculated loss of the swimmers’ velocity is consistent with the experiment. The current simulation can generally represent the glide motion of swimmers.

## Results

Numerical simulations have been conducted for the different initial horizontal velocities, which are 3.1 m/s, 3.3 m/s and 3.5 m/s. The effective research scope of this simulation corresponds to the underwater glide phase of swimmers holding a traditional streamlined posture.

### Swimmers’ trajectories in the glide stage

**Fig 7:**
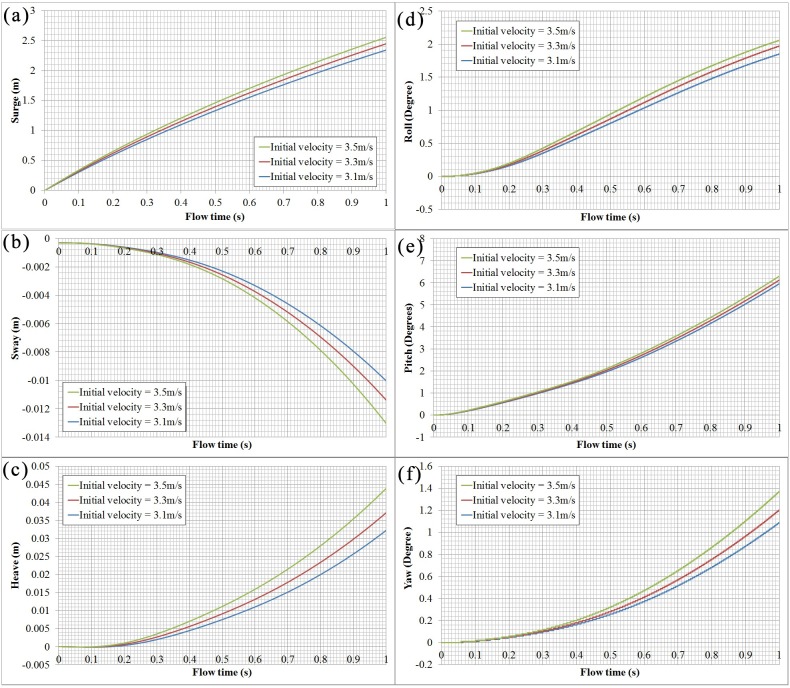
Variation of swimmer’s surge, sway, heave, roll, pitch and yaw with time for different initial velocities.

Fig 7 presents the variation of the swimmers’ six degrees of freedom within the first second of the glide. For different initial velocities, the motion trajectories of the swimmers are similar. Fig 7A shows that the swimmer’s surge value increases monotonically over time. During the glide, the center of the swimmer’s mass shifts slightly to the right as the left-right asymmetry of the swimmer who applies the streamlined posture with hands pronated and overlapping, as shown in Fig 7B. Fig 7C shows that the center of the swimmer’s mass also moves up with gliding forward. Three swimmer’s rotation angles increase monotonically over time, as shown in Fig 7D, 7E and 7F. Particularly, the swimmer’s pitch angle is more sensitive than the other two. The higher the initial velocity, the greater the deviation that takes place from the longitudinal position.

### Hydrodynamic forces

**Fig 8:**
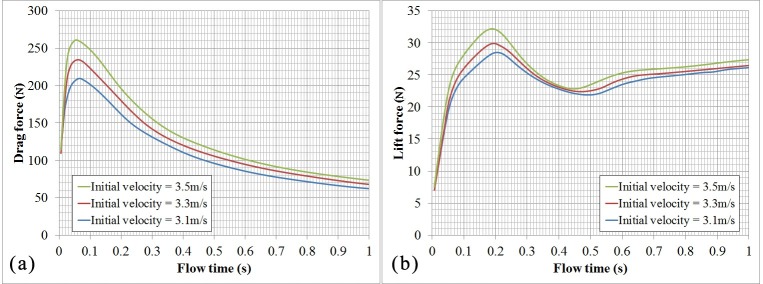
(a) Swimmer’s drag force versus time. (b) Swimmer’s lift force versus time.

Fig 8 presents the variation of swimmers’ drag force with time for swimmers’ surge direction with time at different initial velocities. The swimmer’s drag force increases sharply in the initial phase of the glide as the interaction between the high-speed swimmer’s body and still water [27]. At that time, the swimmer’s velocity is maximal but the velocity of flow field is zero, namely the velocity gradient is maximal at the initial phase, which leads to a performance that the swimmer “shocks” water suddenly. Therefore, the drag force increases sharply at the initial phase of glide. And then the swimmer’s drag force will gradually decrease with time. Similar to the former, there is a sudden increase in the initial phase of the glide for the swimmer’s lift force that has balanced out some of the swimmer’s weight in the vertical direction. However, after an unexpected change at approximately 0.06 s, the swimmer’s lift force continues to increase with a smaller acceleration. The peak of the lift force appears at approximately 0.2 s after gliding. Then, the lift force will gradually decrease until approximately 0.45 s after gliding, and then tends to be stable.

### Visualization of the calculated results

**Fig 9:**
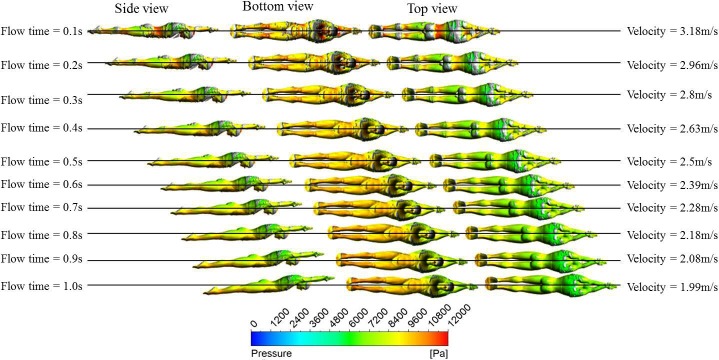
Synthesized map of the variation of swimmer’s distribution and vortex structures (visualized by iso-surface of swirling strength of 17s^-1^) around the swimmer during the glide for initial velocity of 3.5m/s.

For more details, we also visualized the variation of the pressure distribution of the swimmer’s surface and vortex structures around the swimmers during the glide with an initial velocity of 3.5 m/s in Fig 9. During the whole glide stage, the degrees of variation in the high-pressure zone on the swimmer’s surface are more obvious than the low-pressure zone. In the initial glide stage, the high-pressure zones are generated in the depression regions of the swimmer’s body such as the waist, the region between the chin and the chest, the knees and the lower calves. The high-pressure zones will gradually decrease with the loss of the swimmer’s velocity. By contrast, the low-pressure zones are generated at the convex parts of the swimmer’s body, such as the hip and the back. Additionally, we can observe that the large curvature surface of swimming will induce the generation of vortex structures. For example, more abundant vortex structures come into being near the swimmer’s crotch, jaw, chest and back.

## Discussion

The aim of this study was to investigate the glide stage of swimmers using a numerical simulation method, the 6-DOF motion. The swimmers’ displacements, orientations and hydrodynamic forces during the glide are presented and are helpful for optimizing and improving the performance of the swimmers’ glide phase.

The determinant factors affecting glide performance are the initial gliding velocity and the hydrodynamic drag, which decelerate the swimmer [5]. The swimmer’s initial gliding velocity is decided by the preceding actions, including take-off and entry. Hydrodynamic drag is generated during gliding under water. Generally, a swimmer’s hydrodynamic drag can be classified as wave drag, friction drag and force drag. A surface wave is created when a swimmer glides near the water’s surface, and a wave drag is then added to the total resistance. Wave drag can be negligible when the gliding depth exceeds three times the body thickness for general gliding velocities [6]. Lyttle et al.’s study [7] also shows that swimmers might benefit by performing their glides following a turn at 0.4 m underwater to gain maximum drag reduction at velocities above 1.9 m/s. Here, in the present study with a glide depth of 0.9 m, the effect of the wave drag on the swimmer is negligible.

Glide velocity is continually decreased by the hydrodynamic drag, which leads to the variation of the swimmer’s position and angle, thus affecting the glide efficiency. Swimmers’ skin friction drag is due to the shear forces acting on the body surface and can be calculated as for a flat plate at a zero angle-of-attack in terms of the flow characteristic acting on the swimmers’ surface. For a standard swimmer with a general glide velocity, a transition-to-turbulence flow region is presented near the body’s surface [28].During gliding in swimming, the influence of a change of the swimmers’ posture and angle on skin friction is insignificant as far as swimming performance is concerned. When a swimmer travels in water, flow is separated from the swimmer’s surface, which causes a pressure differential throughout the swimmer, and produces form drag on the swimmer. Previous study [29] shows that swimmer’s hydrodynamic drag (especially the pressure drag component) during the underwater gliding could be significantly reduced by wearing a high-performance swimsuit which has features such as ultra-light weight, water repellence, muscles oscillation and skin vibration reduction by the compression effect of body, but this swimsuit was prohibited by the International Swimming Federation (FINA) in 2010 as involving lots of polemics and controversies. The form drag is closely related to the posture and shape of the swimmer. In general, low pressure zones appear in the locations of the swimmer’s jaw, crotch and the wake behind the swimmer for a streamlined posture [19]. Meantime, more abundant vortex structures are presented in those zones. Actually, vortices are important sources of the drag force, and their strength should be reduced as far as possible. Consequently, swimmers can adjust the positions of body parts (e.g., head) to inhibit the generation of vortices for reducing hydrodynamic drag [14]. Additionally, swimmers will gradually deviate from the horizontal position by gliding forward, which causes an increase in the swimmers’ frontal area for motion direction. Swimmers’ total drag force will be continually reduced with the loss of swimmers’ speed, but the reduction rate of swimmers’ total drag force is slowed down to a certain extent as the swimmers’ force area increases.

During the gliding motion, the swimmer’s position is not only influenced by the drag force that acts parallel to the direction of the gliding motion but also by the lift force acting on the swimmer in the vertical direction. In this study, the lift force includes a net force that is created by different pressures on the opposite sides of a swimmer in the vertical direction due to the fluid flow past the swimmer’s body and the buoyancy force that equals the weight of the volume of fluid that is displaced by the swimmer’s body. The calculated lift force that has removed the swimmer’s weight is always positive within the first second of the glide, which explains why the swimmer’s center of gravity has a slight upper displacement in this period. According to the Bernoulli principle, pressure is closely related to the fluid velocity past the swimmer’s body and is inversely proportional to velocity. In the early stages of the glide, obvious low-pressure zones and high-pressure zones are, respectively, generated in the depression regions and convex parts of the swimmer’s body at the relatively high glide speed, so that the maximum lift force appears in this stage. Then, the loss of the swimmer’s velocity acts against the increase in the pressure drag, which causes the decrease of the swimmer’s lift force. However, once the horizontal position changes significantly, flow is reflected, the vertical flow velocity increases, and the lift force gradually increases again in the second half of the glide stage [30].

High coordination between each phase of swimming must occur for high-level competitive swimming. A reasonable instant for starting a leg’s movement after the streamline glide, which makes effective utilization of the advantage of the high-speed glide stage and avoids unnecessary energy consumption for the leg’s movement, is crucially important for improving swimming performance [8]. The swimmer’s velocity decreases between 2.2 and 1.9 m/s, which is considered to be the optimal instant for starting propulsive movement [11]. Starting leg movements too soon will increase the swimmer’s hydrodynamic drag and consume more energy. Starting the leg movements too late will lead to a loss of velocity that impacts the swimming performance.

Traditionally, total start time involves the time from the starting signal to when swimmer arrives at the 15 m mark [31]. The distance of 15 m contains a flight distance of approximately 4 m and the rest distance of 11 m involving the glide with a streamlined posture and underwater leg propulsion. In this study, the numerical results with an initial velocity of 3.5 m/s are implemented to validate the rationality of previous optimal instants for starting leg movement [8, 11]. An assumptive optimal instant to initiate underwater leg propulsion is set as 2.0 m/s that is close to the swimmer’s velocity of underwater leg propulsion, which can avoid the velocity loss as the transition from the glide with streamlined posture to the stable underwater leg propulsion. The assumptive instants for starting leg movement too early and too soon are set as 3.25 m/s and 1.5 m/s, respectively. The velocity loss and the elapsed time of the transition region refer to experimental data by Elipot et al. [10, 11]. Table 2 shows the total time of the start stage for the three different start strategies. The swimmer with the assumptive optimal instant for leg movement is less time-consuming than the other two start strategies. Swimmers will have a larger deviation from the horizontal position that is not favorable for the next stroke if swimmers initiate underwater leg movement too late.

**Table 2:** Comparison of total time for different start strategies.

Instant for initiating leg’s movement	Instant of finishing gliding with streamlined posture			Elapsed time of transition region (s)	Elapsed time of underwater leg’s movement(s)	Elapsed time of flight and diving	Total time (s)
	Glide velocity (m/s)	Swimmer’s sway, heave, roll, pitch and yaw	Elapsed time for gliding with streamline posture (s)				
Leg’s movement too soon	3.25	-0.004m、0.001m、0.2°、0.05°、0.02°	0.09	0.248	4.975	T	T+5.313
Optimal instant	2	-0.14m、0.038m、4.7°、 2.4°、1.3°	1.03	0	4.2	T	T+5.23
Leg’s movement too late	1.5	-0.32m、0.074m、8.1°、 4.25°、2.88°	1.68	0.286	3.4	T	T+5.366

**Fig 10:**
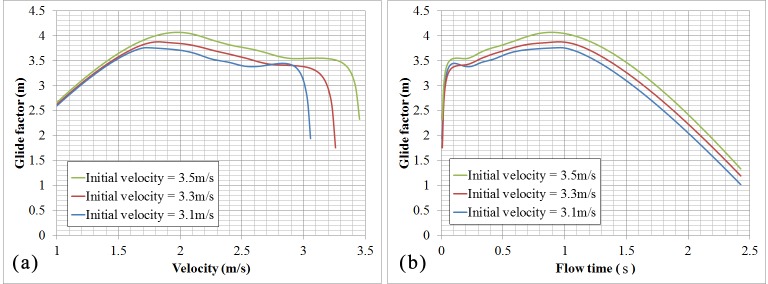
(a) Swimmer’s glide factor versus velocity. (b) Swimmer’s glide factor versus time.

The glide factor, which is a measure of glide efficiency of the swimmer and indicates the ability of the body to minimize deceleration at each corresponding velocity, is can be estimated according to the initial and final velocities and glide interval of a glide. An average glide factor can be defined by following formula:

$$C_{G}=\frac{T}{{\frac{1}{u}}_{x}-\frac{1}{u}}$$ (17)

where *C*_*G*_ is glide factor, *T* is gliding interval duration, *u* is the initial velocity, *u*_*x*_ is final velocity of the glide interval. The higher the glide factor for a gliding body, the smaller the deceleration and therefore the higher the glide efficiency. The rationality of this method has been validated by Naemi and Sanders [3]. From Fig 10, the higher the initial velocity is, the better the gliding efficiency is. A lower glide efficiency appears at that time for initiating leg movements too soon or too late. It is an optimal time for initiating leg movement when the glide efficiency of the swimmers arrives at the maximum value by making the best use of the advantage in glide speed. Calculated results show that an optimal instant for starting propulsive movement is when the swimmer’s velocity decreases in the range of 1.75 m/s to 2.0 m/s, according to the initial horizontal velocity ranging from 3.1 m/s to 3.5 m/s, which is close to the experimental data by Lyttle et al. [7]. Additionally, although swimmers glide with different initial velocities, the optimal instant to initiate leg movement for different initial glide velocities is any time after approximately 0.9–1.0 s of underwater gliding with a streamlined posture.

However, there are some limitations and shortcomings in the current study. This study is based on the assumption that the posture of computational and experimental model remains unchanged which is inconsistent with the facts. Actually, the swimmers may slightly adjust their body to reduce the resistance during the gliding underwater. Besides, this study didn’t take into consideration the swimmers’ water-entry process or the takeoff kick process, which may make a difference to the results.

## Conclusions

This paper has presented a numerical study of the gliding motion of swimmers with different initial velocities based on a 6-DOF full-scale body model. The numerical strategy, where the swimmer’s mass distribution and surface roughness are considered, is proven to be feasible for simulating the underwater gliding motion of swimmer. Numerical results show that swimmers will gradually deviate from the horizontal longitudinal position by gliding forward. The maximum lift force appears at the initial stage of the glide when swimmers glide with a relatively high velocity. Calculated results suggest that an optimal instant for starting propulsive movement is when swimmer’s velocity decreases from 1.75 m/s to 2.0 m/s, according to the initial horizontal velocity of 3.1 m/s to 3.5 m/s. In addition, this study, which first attempts to investigate the glide motions of swimmers using numerical methods, can be a reference that is helpful for people to further understand the gliding stage of swimmers. Some results and discussions are helpful for the swimmers and the coaches, such as when to start propulsive movement, how to reduce the drag force, and so on.
